# Listening Effort Informed Quality of Experience Evaluation

**DOI:** 10.3389/fpsyg.2021.767840

**Published:** 2022-01-05

**Authors:** Pheobe Wenyi Sun, Andrew Hines

**Affiliations:** QxLab, School of Computer Science, University College Dublin, Dublin, Ireland

**Keywords:** Quality of Experience (QoE), cognitive load, listening effort, subjective test, QoE framework

## Abstract

Perceived quality of experience for speech listening is influenced by cognitive processing and can affect a listener's comprehension, engagement and responsiveness. Quality of Experience (QoE) is a paradigm used within the media technology community to assess media quality by linking quantifiable media parameters to perceived quality. The established QoE framework provides a general definition of QoE, categories of possible quality influencing factors, and an identified QoE formation pathway. These assist researchers to implement experiments and to evaluate perceived quality for any applications. The QoE formation pathways in the current framework do not attempt to capture cognitive effort effects and the standard experimental assessments of QoE minimize the influence from cognitive processes. The impact of cognitive processes and how they can be captured within the QoE framework have not been systematically studied by the QoE research community. This article reviews research from the fields of audiology and cognitive science regarding how cognitive processes influence the quality of listening experience. The cognitive listening mechanism theories are compared with the QoE formation mechanism in terms of the quality contributing factors, experience formation pathways, and measures for experience. The review prompts a proposal to integrate mechanisms from audiology and cognitive science into the existing QoE framework in order to properly account for cognitive load in speech listening. The article concludes with a discussion regarding how an extended framework could facilitate measurement of QoE in broader and more realistic application scenarios where cognitive effort is a material consideration.

## 1. Introduction

Quality of experience (QoE) is a paradigm that assesses media quality by mimicking human judgement. The goal is to understand and quantify how consumers perceive media quality. Instead of using the measurable signal parameters, QoE researchers evaluate the quality of a multimedia event based on reported quality ratings from participants in subjective experimental studies. To void the biases from the interpersonal differences, a mean opinion score (MOS) is used to represent an averaged perceived quality. The subjective ratings from experiments are also used to develop signal-based QoE prediction models (also called objective models). Such models are expected to predict quality judgements for multimedia application. Thus, the QoE evaluation approach has been widely adopted to rapidly test the perceptual effect of new products and services.

Despite the wide applicability of QoE evaluation methods, current QoE evaluations for naturalistic multimedia consumption scenarios, when a person is listening to podcasts while driving for example, are limited. They lack the consideration of a person's comprehension, engagement, effort, and other mental status. The current QoE framework, a conceptual model that characterizes how QoE forms, adopts a simple filtering structure that collapse all the interactions of different influencing factors to a single outcome—people's internal comparison between their expectation of the signal properties and what they actually perceive—which can be observed from the subjective quality judgement. Such framework has been widely adopted and works well for many scenarios. For instance, the telecommunication industry uses it to analyse the quality impact of a change in network capacity or system parameters. However, how the cognitive processes affect the multimedia QoE are not addressed by the framework nor by the evaluation methods.

As the multimedia consumption scenarios become more complex, the cognitive aspects of the experience need to be taken into account. QoE evaluation methods applicable to more natural scenarios are important to understand the impact of potential technological changes. Although cognitive aspects are highly personal and are hard to be modeled, the theories and the empirical studies in cognitive science can provide us with practical tools to systematically evaluate the impacts of the cognitive processes. This paper reviews the existing QoE framework as well as the cognitive listening methods and models from the audiology and cognitive psychology domains. The paper then discusses the potential ways to integrate cognitive effort into the existing QoE framework. While this paper uses listening effort as a focus, this review prompts consideration of broader and more realistic QoE framework for application scenarios where cognitive effort is a factor.

## 2. The Existing QoE Framework and Its Limits

### 2.1. The QoE Framework

**Figure 1:**
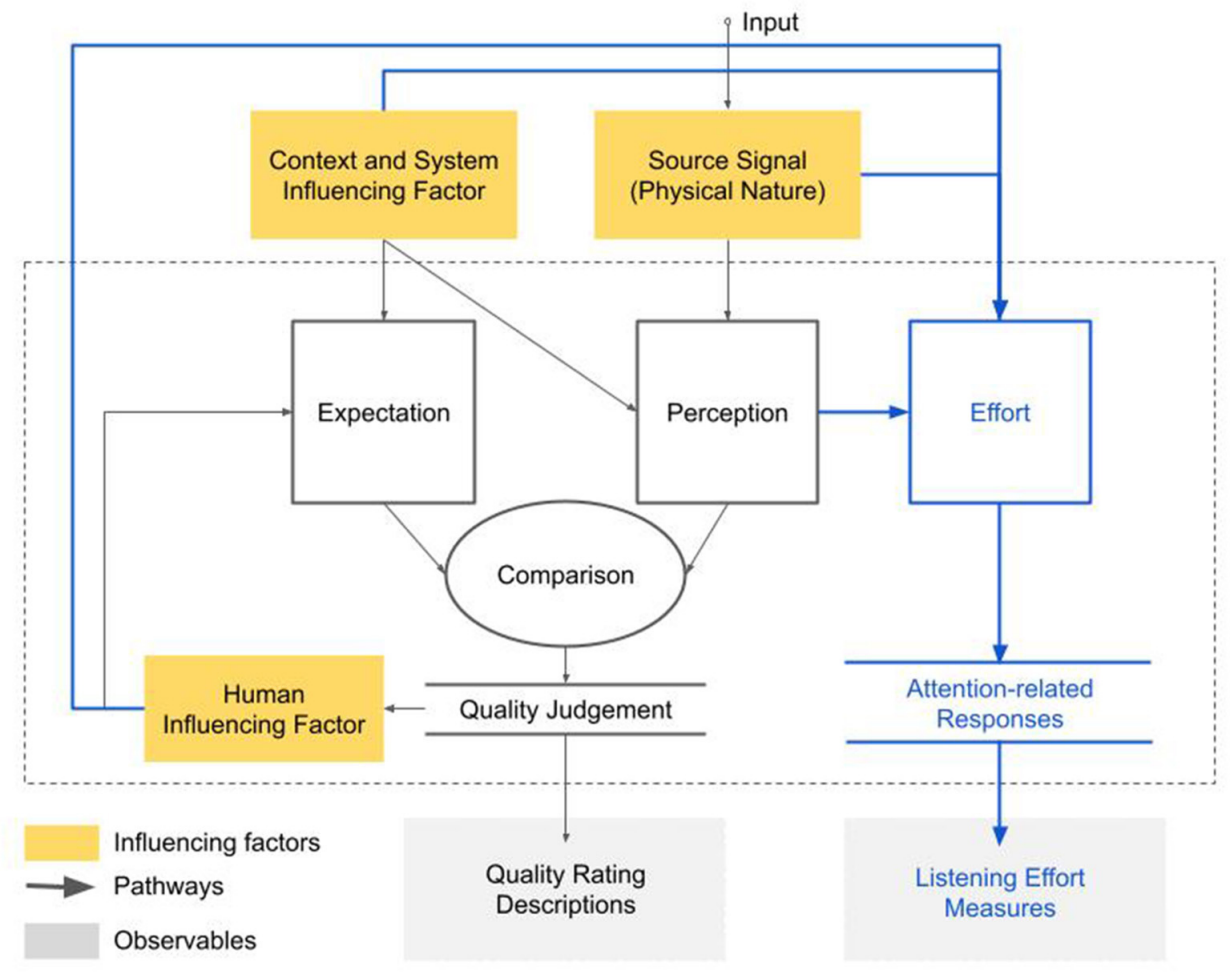
The QoE framework adapted from the QoE whitepaper (Brunnström et al., 2013) where the QoE formation pathways (lines with arrows), the QoE observables (gray boxes), and the QoE influencing factors (orange boxes) are identified. The elements in the existing framework are denoted in black and the expanded parts are in blue. The existing model assumes that the QoE is the outcome of comparing the expected event and the perceived event (see the mechanistic diagrams in black). Both expectation and perception are influenced by different influencing factors. The influencing factors are grouped to four categories (orange boxes). The perceived quality is observed by the subjective rating and/or description of an event (gray box at the bottom).

The QoE framework is a conceptual model that describes a QoE formation mechanism for any multimedia consumption scenario. It can be applied as a template to characterize a quality judgement formation for an experience. The QoE framework identifies the QoE formation pathways, the QoE observables, and the QoE influencing factors (see Figure 1). Quality of Experience (QoE) describes a person's satisfactory level of a perceptual event (Brunnström et al., 2013). It results from the fulfillment of expectations. The satisfactory level of a perceptual experience can be reflected by people's quality judgement. Therefore descriptions and ratings are used as the observables to indicate the latent state of interest—the perceived QoE.

Building on the QoE formation mechanism, *influencing factors* are classified that contribute to either the formation of one's expectation or the perceived event via *formation pathways* (the black lines with arrows in Figure 1). For example, the context of media consumption can influence one's expectation (Sackl et al., 2017), e.g., for a free vs. paid telephone call, or listening-only radio vs. conversational telephone call (Moller et al., 2011). Other factors such as noise and network conditions also affect the perceived event. All the possible QoE influencing factors are grouped to four categories in the QoE framework: signal, context, system, and human factors (Brunnström et al., 2013), each has its own pathway that ultimately contributes to the formation of QoE (see the orange boxes in Figure 1). The identified categories of the QoE influencing factors provide a structural guideline for researchers to analyse the quality impact of any factors of interest in a variety of scenarios. Together with the QoE formation pathways and the observables, researchers can design subjective experimental procedures that yield quantitative QoE measures.

### 2.2. QoE Evaluation in Practice

The two commonly used QoE evaluation approaches, the “descriptive” and the “integrated” (Katz and Nicol, 2019) approaches, conform well with the observables in the QoE framework. The *descriptive* (or *performance*) approach uses the verbal descriptions as QoE evaluation. The focus of the experiential aspects will shift across different application scenarios using this approach. For example, descriptions of the noise and intelligibility levels are useful to evaluate the QoE of a voice call; comments regarding the perceived origin of a sound or how it blends with the rest of the environment are useful in a spatial sound scenario. The *integrated* approach, to the contrary, uses a single numerical value to represent the impression of an overall QoE. For instance, the basic audio quality (BAQ) test (ITU-R, 2015a,b; Schöffler, 2017) uses the mean opinion scores (MOS) for QoE. Using a uni-dimensional representation for QoE makes the comparison of different experiences easier, and hence, making it an efficient solution for rapid evaluations in industry. While acknowledging that experience is a high dimensional concept, the QoE framework provides guidelines to evaluate QoE that is repeatable experimentally and useful for media technology development and evaluation.

### 2.3. The Overlooked Impact of Cognitive Processes

The cognitive processes are modeled in the QoE framework through the pathways connecting the human influencing factors (orange box in bottom left of Figure 1). The human influencing factors comprise factors such as mood, motivation, language, or prior experience (Brunnström et al., 2013). The human influencing factors only contribute to expectation formation, not the downstream QoE formation as human influencing factors are considered to be either temporarily volatile (such as mood and motivation) or personal (such as language proficiency or prior experience). In order to model a QoE evaluation that is representative and relevant for a large population, the effect of the transient factors needs to be dampened in the model. To realize this, QoE evaluation protocols (ITU-T, 1996) recommend implementing a variety of mechanisms to minimize the effect of the human influencing factors such as accent familiarity, voice preferences, fatigue, or boredom. Studies in both audiology and cognitive neuroscience (Pichora-Fuller et al., 2016; Peelle, 2018; Herrmann and Johnsrude, 2020) show that the effort expended on our cognitive process has a substantial impact on perceived experience. Increased listening effort is found to reduce the ability to memorize (Murphy et al., 2000; Rabbitt, 2007; Heinrich et al., 2008; Heinrich and Schneider, 2011), and thereafter comprehension can be adversely affected (Piquado et al., 2012; Ward et al., 2016) due to less context information available from the memory to help decode the current information. A sustained high listening effort is found to lead to lower arousal levels (Aston-Jones and Cohen, 2005) and reduced affective responses (Francis and Love, 2020) such as fatigue (Hockey, 2011) and boredom (Elpidorou, 2018). The strenuous cognitive process is also found to have negative impact on behaviors such as slower response time (Phillips, 2016), inferior task performance (Wingfield et al., 2006; Hornsby, 2013; Lemke and Besser, 2016; Phillips, 2016), or withdrawal from listening task (Lemke and Besser, 2016; Herrmann and Johnsrude, 2020) and social interactions (Mick et al., 2014; Shukla et al., 2020). Several neurological evidences [such as EEG (Hunter and Pisoni, 2018), fMRI (Kuchinsky et al., 2013), and pupil dilation (Aston-Jones and Cohen, 2005; Adank, 2012)] have showed distinct patterns when listeners are exposed to challenging auditory material, indicating the recruitment of different cognitive resources in astute listening scenarios. These findings indicate that the adverse effect of heavy auditory cognition is not only relevant to the population who are diagnosed with hearing impairment, but also relevant to anyone who needs to engage with listening in their day-to-day activities as the recruitment of other cognitive resources can directly affect the allocation of attention and therefore the task performance.

From a multimodal perspective, the existing pathways in the QoE framework are not exhaustive in modeling the effect of different source signals. The combined effect of audio and visual input signals have been shown to produce shifts in attention in various studies (Talsma et al., 2006; Rapela et al., 2012; Chao et al., 2020). Although the multimodal integration is still an active area of study in neuroscience (Koelewijn et al., 2010; Fu et al., 2020), the consideration of audio-visual interaction is shown to be useful for attention and saliency modeling to improve existing QoE prediction (Min et al., 2015, 2020; Zhu et al., 2020).

Attentional saliency, comprehension, fatigue level, task performance, and emotional status are important building blocks for understanding QoE in realistic listening scenarios, and these aspects cannot be captured and fully understood by the quality judgement alone via the standard QoE observable adopted by the community. The existing QoE framework lacks an explicit systematic model to guide effective studies exploring the impact of the cognitive processes on QoE. The attentional control can be influenced by the source signals (e.g., multimodal interaction) as well as by the human influencing factor (e.g., mental capacity). This study will focus on the latter and use the uni-modal input signal as an example to show how studies from cognitive hearing and perception theory could provide complementary learning to supplement the existing QoE framework.

## 3. Integrating Listening Effort Into Existing QoE Framework

To integrate listening effort into the QoE framework model, we consider three questions: (i) what contributes to the increase in the cognitive effort; (ii) how increased effort affects QoE; (iii) how to quantify the effect of effort on QoE. These questions correspond to the three core component in the QoE framework: influencing factors, QoE pathways, and the observables.

This section addresses each question and discuss how each component in the existing QoE framework can be adapted with reference to two cognitive hearing models: the Framework for understanding Effortful Listening (FUEL) (Pichora-Fuller et al., 2016) and the Model of Listening Engagement (MoLE) (Herrmann and Johnsrude, 2020). They also draw on the more general cognitive load models (the load theory Murphy et al., 2016 and the mental capacity model Kahneman, 1973).

### 3.1. Influencing Factors

Listening effort increases along with the listening demand (McGarrigle et al., 2014) as more attentional resources need to be allocated to meet the demand. The FUEL (Pichora-Fuller et al., 2016) model categorizes the sources of listening effort as source, transmission, listener, message, and context factors. These categories all have their counterparts in the QoE framework. Table 1 illustrates how different sources of listening effort can be mapped to different influencing factor categories in the FUEL and the QoE framework. The middle column highlights that all four QoE influencing factor categories contribute to the effort formation. The overlapping factors of concern in both frameworks indicate that the existing QoE framework has already incorporated the main factors that lead to listening effort. The next step is to analyse whether the cognitive effect of these influencing factors can be modeled by the QoE formation pathways.

**Table 1:** Sources of listening effort and their corresponding influencing factor categories in the QoE framework and the FUEL.

**Factors**	**QoE**	**FUEL**
Voice degradation	System	Transmission
Bandwidth limit	System	Transmission
Noise	System	Transmission
Reverberation	System	Transmission
Multi-talker	Signal	Source & context
Spatial separation	Signal	Source & context
Synthesized voice	Signal	Source
Sustained speech	Context	Source
Voice similarity	Signal	Source
Foreign language	Signal & context	Message & context
Reward	Human	Motivation
Hearing loss	Human	Listener

### 3.2. Pathways

The formation pathways in a model identify the possible mechanisms through which the influencing factors can follow to impact an outcome. Although the formation pathways are not concrete, they are depicted in the models to guide research protocol designs wishing to evaluate the effect of factors of interest. The implications of increased listening effort are the result of complex combinations of interactions. The existing QoE formation pathways collapse the contributions of influencing factors to an internal comparison, which limits the capacity to capture the wider cognitive effects that make up our listening experience. Cognitive hearing studies (McGarrigle et al., 2014; Pichora-Fuller et al., 2016; Herrmann and Johnsrude, 2020) indicate that multiple effort formation pathways exist during speech listening. When a speech signal is being processed at an early stage, with presence of noise for instance, effort arises when listeners inhibit the irrelevant signals and keep attentive to the target signals. However, sometimes a higher load level helps people to concentrate (Mick et al., 2014; Murphy et al., 2016; Herrmann and Johnsrude, 2020). At a later stage when the speech signal is being processed semantically, effort increases when the content topic is obscure and more context information needs to be recalled from memory to aid comprehension. Effort is also be influenced by the demands of concurrent tasks (Skowronek and Raake, 2014) as attention needs to be constantly reallocated depending on the dynamics of a subtask. This pathway is particularly relevant to the design of technology and multimedia applications where people increasingly consume multimedia while multi-tasking in day-to-day scenarios.

It has yet to be shown whether the effect of multiple effort formation pathways can be simplified to a single pathway. Therefore, we show multiple potential effort formation pathways so that systematic investigations into the cognitive impact can be designed. Multiple pathways might result in different experiential implications in addition to the quality judgement, thus additional measurements that capture different aspects of an experience need to be recorded to compare the differences in the perceptual experiences.

### 3.3. Observables

The observables are used by researchers to infer the impact of influencing factors. The choice of the observables depends on the outcome of interest and the corresponding formation pathways. For instance, the corresponding observables for the percept (Johnsrude and Rodd, 2016), cognitive activity, and the mental capacity as a result of listening effort can be the self-reported responses, neuroimaging, and concurrent task performance. As multiple listening effort formation pathways might exist, a single observable (i.e., a quality judgement) may not be sufficient to capture the QoE. Initiatives in the QoE domain (Engelke et al., 2017) already attempt to use other observables to give a broader definition of QoE. We will next summarize the various listening effort observables in use and discuss how different types of observable account for different aspects of an experience.

The most direct observables for listening effort are the self-reported ratings or descriptions. Ratings are more commonly adopted as they are both scalable and easier to process. The NASA-TLX mental effort scale (Hart and Staveland, 1988), for example, is a mature instrument that asks subjects to rate on different relevant aspects such as fatigue, stress, and task difficulty to gauge one's overall cognitive load (Rubio et al., 2004). Another example of a self-reported measure asks subjects to estimate the duration they can sustain a task to gauge the cognitive load while listening (Pichora-Fuller et al., 2016). However, due to the retrospective nature of these self-reported measures, such measures are susceptible to memory and descriptive biases.

Behavioral responses are also used to indicate effort. These include the memory recall, speech comprehension (observed after the task), or attention-related task performance (observed during the task). The Span Test (Conway et al., 2005) is a well established working memory test where participants are asked to read a series of sentences and to recall the last word from each sentence. It is used to indirectly evaluate listening effort based on the assumption of working memory capacity (Baddeley, 2000). In a demanding listening scenario, an increase in the allocated cognitive resources to comprehend the signal will adversely impact information recall capacity. Another popular experimental paradigm is the dual-task method where participants conduct a parallel task simultaneously to force the division of attention. In this case, an increase in the listening effort is indicated by a performance reduction in the concurrent task (Hunter, 2020). The dual-task paradigm is based on the assumption that attention allocated to one task will leave less spare cognitive capacity to process another task (Kahneman, 1973; Beatty, 1977; Sweller, 1994; Schnotz and Kürschner, 2007) leading to an observable reduced performances in the less attended task.

Psychophysiological changes are also used to indicate the effort involved in a listening task. Some physiological observables (e.g., pupil dilation, cardiac responses, skin conductance, and hormonal changes) are the result of sympathetic or parasympathetic responses to stress or effort (de Waard, 1996; Peelle, 2018). Thus, they are regarded as indirect measures for listening effort. Observables captured around the brain area (such as the activity intensity and the differences in the activated brain regions) are also used as indicators of listening effort. For example, an increase in the alpha band power in the electroencephalography signal can be observed when there is signal degradation or an increased demand for information storage (Piquado et al., 2012; Pichora-Fuller et al., 2016; Hunter, 2020). An increase in activity is found in the cingulo-opercular network from the functional magnetic resonance imaging when listeners are exposed to less intelligible signals (Wild et al., 2012; Erb et al., 2013; Vaden et al., 2013; Eckert et al., 2016). The psychophysiological observables are highly susceptible to many other internal and external factors such as environment temperature and mental status. Yet the high resolution in time makes them the preferred instruments for event-related analysis.

Identifying the potential and appropriate observables is critical in order to select the methods that will capture how effort affects different aspects of our experience. Using multiple observables is also recommended to reduce the structural interference in data analysis (Kahneman, 1973; Pichora-Fuller et al., 2016). The theoretical and empirical cognitive psychology literature provides a broad selection of observables to complement the commonly-used self-reported measures in the QoE community. It also prompts looking beyond the existing QoE framework to consider pathways to better capture different impacts of listening effort in naturalistic scenarios.

## 4. Conclusion and Future Direction

This review introduced the QoE framework model used by the media technology community to assign in designing and selecting the appropriate methods to empirically evaluate quality of experience. We introduced the rationale behind the framework and explained the structural influencing factors, pathways and observables. The limited capability within the framework to capture and quantify how effort interacts with QoE was highlighted. With a focus on listening effort, this paper reviewed multiple listening effort formation pathways from the cognitive science domain to complement the existing QoE formation pathway. A review of literature and methods drawn from the audiology and cognitive science domains, illustrated how the QoE framework could be expanded and QoE experimental methods could be applied to naturalistic listening scenarios where the cognitive process plays a significant part in QoE formation. Pathways and observables beyond self-reported quality ratings were reviewed. We believe the review warrants adding a cognitive dimension to QoE framework. It would allow for more direct comparisons of different subjective experiments. It would encourage the community to design subjective experiments that consider the impact of less explored cognitive processes. Furthermore, subjective experiments guided by such framework should provide new insights into the more nuanced experiential aspects of our multimedia consumption experience.

More generally, the review highlights the flexibility within the framework for extension and the potential to capture a better understanding of audio influence within wider QoE studies, e.g., listening effort impacting video or immersive QoE. This review also presents an opportunity to apply a similar approach beyond listening, identifying new pathways and observables within the QoE framework, for visual, haptic or multimodal interactions.

## Author Contributions

PS and AH both contributed to writing, development, and editing. Both authors contributed to the article and approved the submitted version.

## Funding

This publication has emanated from research conducted with the financial support of Science Foundation Ireland (SFI) under Grant Number 12/RC/2289_P2.

## Conflict of Interest

The authors declare that the research was conducted in the absence of any commercial or financial relationships that could be construed as a potential conflict of interest.

## Publisher's Note

All claims expressed in this article are solely those of the authors and do not necessarily represent those of their affiliated organizations, or those of the publisher, the editors and the reviewers. Any product that may be evaluated in this article, or claim that may be made by its manufacturer, is not guaranteed or endorsed by the publisher.
